# Clinicopathologic and genomic characterizations of brain metastases using a comprehensive genomic panel

**DOI:** 10.3389/fmed.2022.947456

**Published:** 2022-11-24

**Authors:** Duna H. Barakeh, Ebtehal Alsolme, Fatimah Alqubaishi, Amal Almutairi, Lamees Alhabeeb, Sally Al Abdulmohsen, Shahd S. Almohsen, Doaa Alayed, Sara Rashid AlAnazi, Malak AlZahrani, Albandari Mohammed Binowayn, Sarah S. AlOtaibi, Fahad A. Alkhureeb, Wafa Al Shakweer, Hindi Al-Hindi, Ali Alassiri, Heather A. Robinson, Malak Abedalthagafi

**Affiliations:** ^1^Department of Pathology, King Khalid University Hospital, King Saud University, Riyadh, Saudi Arabia; ^2^Genomics Research Department, King Fahad Medical City, Riyadh, Saudi Arabia; ^3^Department of Pathology, King Fahad Medical City, Riyadh, Saudi Arabia; ^4^Department of Pathology and Laboratory Medicine, King Abdulaziz Medical City, Riyadh, Saudi Arabia; ^5^Department of Pathology, Security Forces Hospital, Riyadh, Saudi Arabia; ^6^Department of Pathology, King Faisal Specialist Hospital and Research Center, Riyadh, Saudi Arabia; ^7^Health Research, University of Manchester, Manchester, United Kingdom; ^8^Department of Pathology and Laboratory Medicine, Emory University Hospital, Atlanta, GA, United States

**Keywords:** PI3K, CDK, breast cancer, brain metastasis, comprehensive genomic panel, colorectal cancer, genetics diversity

## Abstract

Central nervous system (CNS) metastasis is the most common brain tumor type in adults. Compared to their primary tumors, these metastases undergo a variety of genetic changes to be able to survive and thrive in the complex tissue microenvironment of the brain. In clinical settings, the majority of traditional chemotherapies have shown limited efficacy against CNS metastases. However, the discovery of potential driver mutations, and the development of drugs specifically targeting affected signaling pathways, could change the treatment landscape of CNS metastasis. Genetic studies of brain tumors have so far focused mainly on common cancers in western populations. In this study, we performed Next Generation Sequencing (NGS) on 50 pairs of primary tumors, including but not limited to colorectal, breast, renal and thyroid tumors, along with their brain metastatic tumor tissue counterparts, from three different local tertiary centers in Saudi Arabia. We identified potentially clinically relevant mutations in brain metastases that were not detected in corresponding primary tumors, including mutations in the PI3K, CDK, and MAPK pathways. These data highlight the differences between primary cancers and brain metastases and the importance of acquiring and analyzing brain metastatic samples for further clinical management.

## Introduction

Brain metastases constitute the majority of intracranial cancers, and are often associated with a poor prognosis. Brain metastasis is a major healthcare burden, especially involving cancer patients with recurrences. Classic treatment modalities usually include radiation and supportive treatments, including glucocorticoids among others (1). With the evolution of neurosurgery, these patients can also be treated with surgery, stereotactic radiosurgery (SRS), and fractionated stereotactic radiotherapy (FSRT) (2). The gold standard for diagnosis is radiology through Computed Tomography (CT) scans. In very few cases, these patients undergo an excisional biopsy (which can be curative of some signs and symptoms), or even CSF fluid aspiration (3). Such specimens allow the retrieval of the concentrated DNA of tumor cells via a cascade of steps needed for Next Generation Sequencing NGS and other helpful tumor analysis techniques (4).

The ability of cancer cells to metastasize has been attributed to the migratory and invasive capabilities of cancer cells, which depend on cell-to-cell interactions and communication with the cellular and extracellular matrix microenvironment, in addition to immune reactions and genetic factors (5–7). The most common location for a tumor metastasis varies from one primary tumor type to another, with various transmitting routes such as arteries, the lymphatic system and/or direct extension (8). The most common locations for metastasis are the lung and liver, with the incidence of brain metastasis approaching 1.9% for gastric cancer (9).

For tumor cells to cross the blood brain barrier (BBB), extravasation, migration, adhesion and proteolysis are required, in addition to changes in the brain-specific tumor microenvironment (TME), in order to accommodate subsequent metastatic tumor growth (10), which often is more aggressive compared to the primary tumor (11). Tumor types including breast cancer, colorectal and thyroid derive their metastatic potential from cadherins, TP53 loss, and other proteolytic enzymes and soluble molecules involved in tumor epigenetics, which may help in them crossing barricaded BBB defenses through the endothelial cell layer of brain capillaries and vessels (10).

Previous studies have demonstrated genetic heterogeneity between metastatic and primary tumor pairs (12, 13). Genetic profiling of brain metastatic tumors and their primary tumor has identified the activation of the PI3K and HER2/EGFR pathways, amongst others, in the metastasizing tumor (14). As brain metastasis is considered an ominous sign that might resign some patients to palliative instead of curative therapy, we aimed to perform a study targeting metastasizing tumor cells found in the brain. However, due to re-sampling difficulty in such patients, the sample size remained too low to predict a generalized approach. The genetic heterogeneity of metastases makes resampling challenging, since another sample from the same metastasis might have a different genetic make-up (15, 16).

A better knowledge of targetable signaling pathways in specific metastases may shift current routine therapies toward more targeted therapeutic approaches (16, 17). Importantly, there is a need to study the genetic drivers in non-western populations, to ensure scientifically sound treatment therapies based on specific genetic changes in these populations. Therefore, we performed Next Generation Sequencing (NGS) on 50 pairs of primary tumors including but not limited to: colorectal, breast, renal, and thyroid tumors with their brain metastatic tumor tissue counterparts, from three different local tertiary centers providing full clinical care for our cohort. Our results show a unique pathway-specific activating mutation pattern in brain metastases in comparison to their corresponding primary tumors, thus highlighting the importance of studies like ours to develop therapeutic strategies targeting metastasis-specific pathways.

## Materials and methods

Tissue samples were collected from 50 Saudi patients at three different local tertiary centers diagnosed with metastatic brain tumors in the period of 2010–2021 (King Fahad Medical City, King Abdulaziz Medical City, and Security Forces Hospital-Riyadh). IRB approval was obtained from all participating centers.

Samples were collected as FFPE (Formalin Fixed Paraffin Embedded) tumor blocks with a tumor percentage of >90%. DNA was manually extracted from the blocks using the GeneRead*™* DNA FFPE Kit (QIAGEN). We performed the NGS panel of Oncomine Comprehensive v3–w4.2 DNA–Single Sample. This panel detects and annotates low frequency somatic variants (SNPs, InDels, CNVs) from targeted DNA libraries from the Oncomine Comprehensive Assay panel v3 (OCAv3, Thermo Fisher Scientific, Waltham, MA, USA) run on the 540 chips. Released with: Ion Reporter Software 5.18. Workflow Version: 4.2 (18).

### Statistical analysis

Descriptive statistics including means, standard deviations, medians, IQRs (interquartile ranges), and frequencies were obtained for age at primary tumor and brain metastasis diagnosis, and metastatic free interval (MFI- difference between primary and metastatic diagnosis dates), the only quantitative variables considered. A Cox proportional hazards model was then constructed using R statistical software (v4.0.5), to assess the relationship between survival to the end of the study period by age at brain metastasis and the following predictive variables: gender, MFI, having anti-HER2 treatment prior to brain metastasis (breast cases only), having chemotherapy prior to brain metastasis, having targeted therapy prior to brain metastasis, having nodal involvement and primary tumor grade. Smoking was omitted from the model due to the small number of smokers included (*n* = 3). Where the date of primary diagnosis was missing but the age at primary diagnosis was known, the date of primary diagnosis was estimated as the date at the median of the potential interval.

Kaplan-Meier curves were created to visualize survival by significant independent categorical predictors indicated by the Cox survival model. A log-rank test was carried out for each pair of Kaplan Meier curves to determine the chi squared and *p*-value for the difference between groups, using the R survdiff function. The R pwr package was used to assess the power of the sample size at various effect sizes, and to determine the limitations of the analysis.

## Results

### Clinical and histopathological findings

**FIGURE 1:**
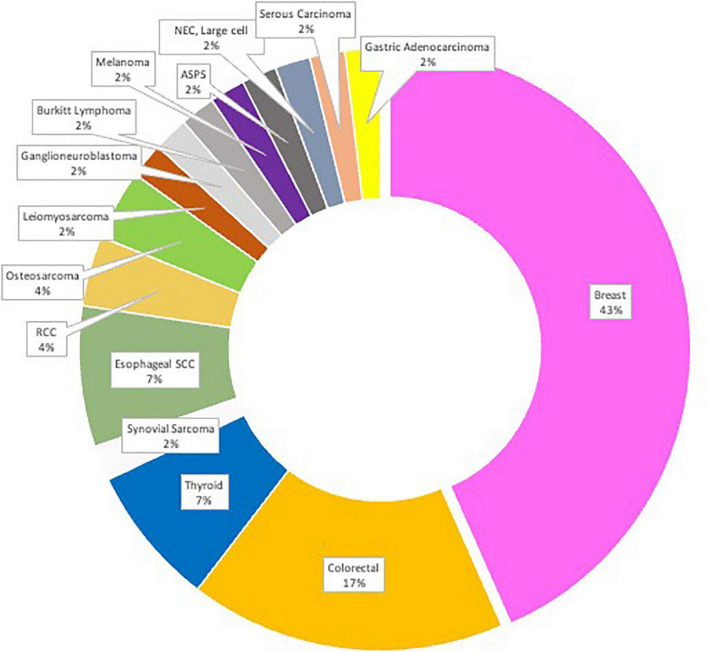
The study cohort (*N* = 50) distribution by location of primary tumor with corresponding metastatic CNS tumor(s).

Our patients included 31 males and 19 females, with a mean age of 49 and ages ranging from 11–83 years (clinical characteristics summarized in Table 1). All centers are tertiary referral oncology centers. All cases were diagnosed as primary and metastatic brain tumors with histopathological diagnoses including the following tumors: Alveolar Soft Part Sarcoma (ASPS), Burkitt Lymphoma, Colorectal Carcinoma (CRC), Ganglioneuroblastomas, Gastric adenocarcinoma, Invasive Ductal Carcinoma (IDC), Invasive Lobular Carcinoma (ILC), Leiomyosarcoma, Melanoma, Neuroendocrine Carcinoma (NEC) large-cell type, Osteosarcoma, Ovarian Serous Carcinoma, Clear Cell and Papillary Renal Cell Carcinoma (RCC), Keratinizing and Non-Keratinizing Squamous Cell Carcinoma (SCC), Papillary, Poorly-differentiated, and Anaplastic Thyroid Carcinomas. Numbers of cases for each tumor type are summarized in Figure 1. Primary tumors were diagnosed between 2005 and 2020. The most common tumor types observed are reflective of the availability of both primary and metastatic brain tumors found in the centers involved.

**TABLE 1:** Patient clinical characteristic summary (categorical variables).

Clinical characteristics	*N* (*n* missing)	Mean [SD]	Median (IQR)
All patients	50		
**Gender**			
Female	31		
Male	19		
**Age (years)**			
Age at primary tumor diagnosis		44.1 []	43 (34.0, 54.8)
Age at brain metastasis diagnosis		46.5 []	46 (36.5, 57.0)
Metastasis free interval		2.3 []	1.5 (0.73, 2.55)
**Other variables**			
Chemotherapy prior to brain metastasis	39 (2)		
Targeted therapy prior to brain metastasis	15 (2)		
Immunotherapy prior to brain metastasis	9 (3)		
Brain radiation prior to metastasis resection	1		
Smoking history	3		
H/O metastasis at another site	38		
Primary tumor histological grade: High	21 (15)		
Primary tumor histological grade: Low	14 (15)		
Nodal involvement	24		
**Therapy combination for primary tumor**			
Chemotherapy only	20		
Chemotherapy, immunotherapy	2		
Chemotherapy, targeted, anti-HER2	8		
Chemotherapy, immunotherapy, targeted	1		
Chemotherapy, anti-HER2	1		
Chemotherapy, targeted	2		
All of the above	4		
Immunotherapy only	2		
None of the above	7		
Unknown	3		
**Breast primaries total**	22		
IDC	20		
ILC2	2		
Anti-HER2 treatment prior to brain metastasis	13		
Positive HER2 status for primary tumor	11		
Positive HER2 status for metastasis	10		
Triple negative primary	6		

We studied patient outcomes by analyzing age at metastasis, MFI and disease-free survival, in addition to immunotherapy and radiotherapy treatments applied to these patients. In total, 15 patients were lost to follow-up, leaving 35 with known survival status at the end of the study period.

As would be expected for patients with brain metastasis, almost half of the patients presented with a high grade and nodal metastasis, and 38 patients had a history of metastasis at other sites (Table 1). The majority of the patients (39 cases) had received chemotherapy prior to brain metastasis, while nine patients had received immunotherapy and one patient received radiotherapy prior to brain resection. Only three patients in our cohort were smokers, therefore any effect of smoking could not be analyzed.

Focusing on our 22 breast cancer samples, 13 patients had received Anti-HER2 ERBB2 (Human Epidermal Growth Factor Receptor 2) gene therapy, despite only 11 showing HER2 ERBB2 positivity in their primary tumor samples, and ten of these showing the same positivity in their metastatic tumors.

### Next generation sequencing analysis

**FIGURE 2:**
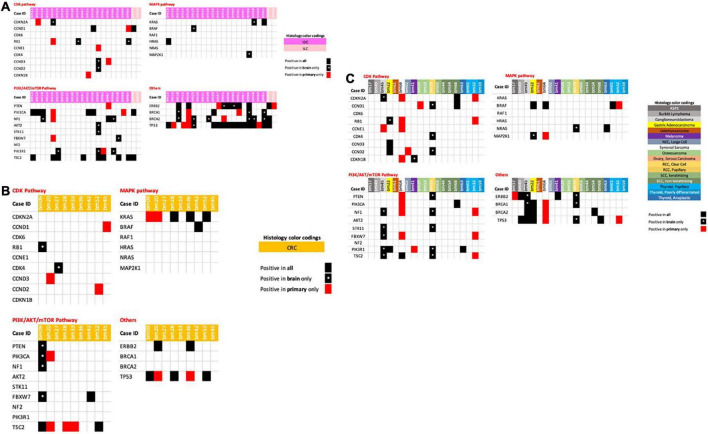
**(A)** Breast cancer primary and metastatic tumors with associated genetic mutations. **(B)** Colorectal cancer primary and metastatic tumors with associated genetic mutations. **(C)** Miscellaneous/other tumors primaries and metastatic tumors with associated genetic mutations.

Each primary tumor and its metastatic counterpart showed increases in the number of genomic aberrations. Some tumors showed different mutations, fusions, SNVs, CNVs, indels, and copy number aberrations within the same gene, while others showed new mutations in otherwise wild-type primary tumors. We summarize the most important findings in Figures 2A–C, with a special focus on the genetic markers for common and established targeted therapies.

We focused on the availability/absence of Cyclin Dependent Kinase pathway (CDK)-associated genes such as *CCND1, CCND2, CCND3*, *CCNE1, CDKN2A, CDK6, CDK4, CDKN1B*, and *RB1* (Retinoblastoma 1), in addition to Mitogen Activated Protein Kinase (MAP-K) pathway-associated genes including *KRAS* (Kirsten rat sarcoma virus), *BRAF* (v-Raf murine sarcoma viral oncogene homolog B), *NRAS* (Neuroblastoma RAS viral [v-ras] oncogene homolog), *HRAS* (Harvey Rat sarcoma virus) and *MAP2K1*. Another signaling cascade we focused on is the PI3K-AKT-mTOR pathway involving phosphoinositide 3-kinase (PI3K), protein kinase B (Akt) and the mammalian target of rapamycin (mTOR), with its associated genes *PIK3CA, PIK3R1, NF1*, and *NF2* (Neurofibroma genes), *AKT2, PTEN* (Phosphatase and TENsin homolog), FBXW7 (F-Box and WD Repeat Domain Containing-7), and *TSC2* (Tuberous sclerosis complex). Other highlighted genes include *ERBB2*, Breast Cancer Genes 1 and 2 (*BRCA1* and *BRCA2*) and *TP53* (tumor suppressor protein p53).

When analyzing breast cancer tumors, including IDCs and ILCs, we observed new mutations in *CDKN2A, RB1, CCND2*, and *CCND3* in the brain metastases of four IDCs, but not in their primary tumor counterparts (Figure 2A). Other mutations of *CCNE1* were detected in both tumors in each case (primary and brain metastasis), while six primary IDC tumors showed mutations that were not present in the brain metastatic counterparts.

When analyzing the PIK/AKR/mTOR pathway, we detected the same mutations for both primary tumors and brain metastases in 11 patients. One patient had a *PIK3CA* mutation in the primary tumor but not in the brain metastasis, and five patients harbored new mutations in their brain metastasis tumors only. These results might indicate that these patients could benefit from therapies targeting the PI3K/AKT/mTOR pathway.

The MAPK pathway showed mutations in the *BRAF, KRAS*, and *MAP2K1* genes in three brain metastatic tumors that were not present in their breast primary tumors. Mutations in *BRCA1* and *BRCA2*, in addition to *ERBB2*, were also newly present in brain metastatic tumors and not detected in primary breast tumors in eight out of 22 breast cancer patients. Novel mutations in *TP53* were only present in one metastasis sample.

Considering colorectal cancer (CRC) samples, one patient had mutations in *RB1, PTEN, PIK3CA, NF1*, and *FBXW7* in one brain metastasis, but not in the primary tumor, while another patient showed mutations in *CDK4* only, indicating that these patients might benefit from CDK and PIK/AKT/mTOR pathway inhibitors (Figure 2B). Other CRC patients also highlighted the need to re-assess their brain metastatic tumors through mutational analysis of the *CCNDs, KRAS* and *TSC* genes, with loss of the mutation previously also seen for TP53 (Figure 2B).

Other tumors in this cohort are shown in Figure 2C, enabling us to identify new mutations in components of the PIK/AKT/mTOR pathway in brain metastasis tumors of primary ganglioneuroblastoma. We also identified new mutations in the CDK and PI3K/AKT/mTOR pathways in clear cell RCC brain metastasis tumors, while a melanoma brain metastasis also showed a single new CDKN1B gene mutation, suggesting the potential for CDK inhibitor therapy in these patients.

Our coverage of all potential mutations will not be comprehensive, as neither a synovial primary sarcoma nor its brain metastasis showed mutations in any of the genes analyzed here. Similarly, no mutations were found in the MAPK, CDK, and PI3K/AKT/mTOR pathways in thyroid tumors and their brain metastases, which would suggest that a therapy targeting these pathways would not be clinically recommended.

### Survival analysis

**FIGURE 3:**
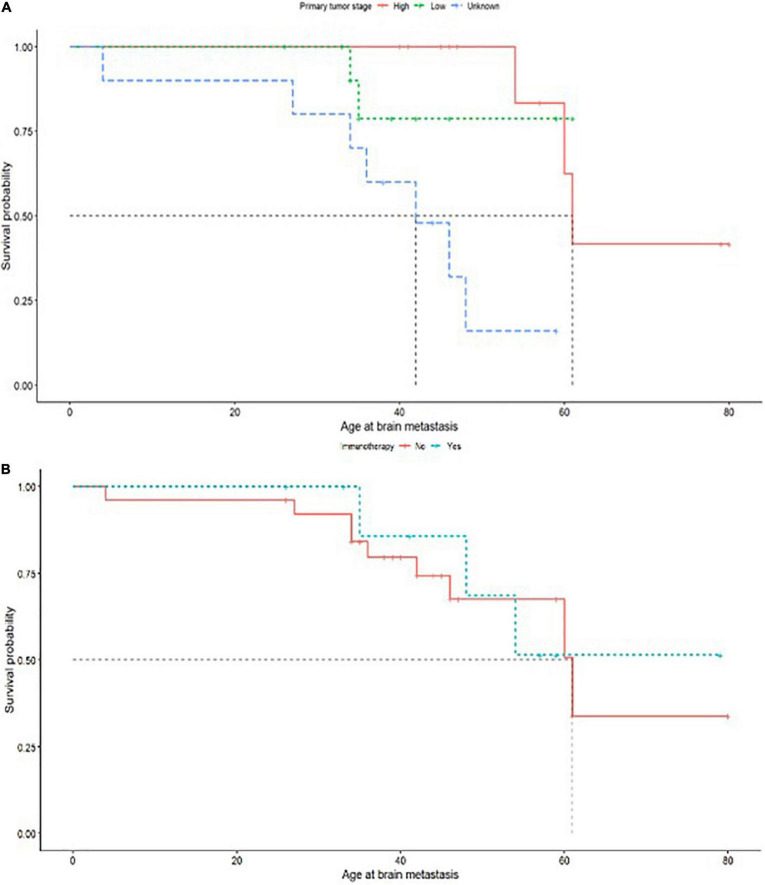
**(A)** Primary tumor stage of high or low with survival probability related to age of brain metastasis. **(B)** Immunotherapy treatment administered as (yes/no) with survival probability related to age of brain metastasis.

Log rank comparisons of Kaplan Meier curves comparing survival by age at metastasis by levels of each predictor variable did not flag significant relationships between these variables and survival (the corresponding *p*-values did not reach the <0.05 threshold for significance). However, a Cox proportional hazards model was used to determine predictors significantly associated with survival by age at brain metastasis, showing a significant negative association between survival and primary tumor stage; Death during the study window adjusted for metastatic age was disproportionately likely if the primary tumor stage was unknown (*p* = 0.03) which may reflect late diagnosis of a primary tumor close to the diagnosis date of a metastatic brain tumor. A Kaplan Meier plot for the univariate relationship is shown in Figure 3A (Log Rank test *p* = 0.002). There was also a significant positive relationship between the use of immunotherapy for primary tumor treatment and hazard of death in brain metastatic patients (*p* = 0.03, Figure 3B), most likely because of association of this treatment pathway with specific forms of cancer which have a poorer prognosis. Log rank comparison suggested differences in survival curves with immunotherapy were not significant (*p* = 0.8) (Figure 3).

Although some clinical history predictors did not independently predict survival in this cohort which may have been expected to do so, including prior treatment type, it may be that primary tumor stage resamples these variables by acting as a proxy for overall primary tumor severity, which correlates with tumor size and site and also influences treatment choice. Sixteen patients received a combination of multiple treatments for their primary tumor (Table 1). A power calculation indicated that in a cohort of 35 (with survival outcomes recorded) we would be able to detect effect sizes over 0.5 at 88% confidence, but may have missed smaller effects (42% likelihood of detecting significance where effect size = 0.3). Correlations which approached but did not reach significance in this study included lymph node involvement in primary tumors (*p* = 0.08) (Figure 3).

The Cox proportional hazards model and the Kaplan-Meier curves will be to some extend skewed by left censoring, as the actual age at brain metastasis will typically be younger than the observed age at brain metastasis diagnosis, dependent on screening interval. We make an assumption that this screening interval is approximately equivalent for patients with similar diagnoses, and therefore anticipate that the same basic time trends will be seen for survival, albeit over a longer time interval.

## Discussion

This study compared mutational burdens in a wide spectrum of primary tumors including breast cancer, colorectal cancer, melanoma, synovial sarcoma, gangioneuroblastoma, renal cell carcinoma and thyroid cancer, and their associated brain metastases. We hypothesized that specific signaling pathways might be affected by mutations in the pathway components of brain metastases, but not the original primary tumor. Our results show metastasis-specific mutations in a number of pathways and cancer types, including the MAPK, CDK, PIK/AKR/mTOR, and other pathways in breast cancer patients.

Previous literature has highlighted the need to explore the PI3K/AKT/mTOR pathway in aggressive tumors (19), and this includes brain metastatic tumors and their primary counterparts, suggesting the PI3K/AKT/mTOR pathway as a potential therapy target to prevent brain metastasis formation (20, 21). PI3K/AKT/mTOR inhibitory agents that have been studied in literature, such as rapamycin and everolimus, which both inhibit mTOR, are expected to lead to better therapeutic responses compared to traditional treatment modalities in primary breast cancers (22). Moreover, primary renal cell carcinomas, melanomas and neuroendocrine tumors appear to respond well to promising potential pathway inhibitory therapies of the mTOR pathway such as everolimus (22). Angiogenesis problems and treatment resistance have been associated with targeting the mTOR pathway (21, 23).

Similar findings of a need for regular treatment strategy review were identified in targeted CDK therapies for breast cancer patients with metastases utilizing CDK4/6 inhibitors (24). Positive outcomes are seen in situations where certain CDK4 inhibitory agents have the ability to cross the blood brain barrier (BBB) (25). The development of suitable agents is still underway, but survival is observed to improve when using the CDK inhibitor Abemaciclib combined with radiation therapy, in studies focusing on breast cancer brain metastases (26, 27). In addition, the CDK4 pathway has been newly mutated in our brain metastasis samples of Clear cell RCCs and not in primary tumor cells, while tumors of neuroendocrine carcinoma, synovial sarcoma and keratinizing SCCs show mutations only in their primaries. Synovial Sarcomas harboring CD4 mutations in literature were correlated with higher stages—and hence more propensity for brain metastasis—which gives hopes for potential targeted therapies exploiting their apoptotic activity and administering agents such as palbociclib (26, 28). Other CDK4 inhibitory agents such as ribociclib have been shown to affect RCCs also (29).

Our analysis of genes involved in the MAPK pathway did not show any activating mutations that would lead to pathway activation in the analyzed tumors (Figures 2A–C). Very few breast cancer tumors were positive for mutations in the MAPK pathway, and our single melanoma patient showed no mutations in this pathway, which might be due to the lack of a sufficient sample size. Previous studies showed efficacy of targeting the MAPK pathway in the treatment of metastatic melanoma and other tumors (30–32). We do see MAPK pathway genes mutations involving our gastric adenocarcinoma and clear cell RCC tumors in the brain and not in primary tumors. This has been further studied in Asian populations where gastric adenocarcinomas persist (33). MAPK pathway inhibitors have been seen to harbor a potential role in treating RCCs (34).

Common potential therapeutic target genes such as *BRCA1/2*, *TP53*, and EGFRs were positive in our metastatic samples of breast cancer and renal cell tumors but not in primary tumors (Figures 2A,C). Novel mutations in these genes in brain metastatic tumors have been reported in previous studies, making the targeting of these genes a promising therapeutic approach to prevent or halt brain metastases in cancer patients (4, 35).

Cancer cells utilize multiple mechanisms including migration, invasion, extravasation and intravasation, to navigate through multidimensional environments in order to travel to and populate the metastatic target organ (36). The brain microenvironment poses a complex environment for these cancer cells that requires adaptations. These adaptations include epigenetic changes that control gene expression levels rather than protein coding sequences, in addition to new somatic genetic mutations observed in metastatic but not primary tumors (36, 37). In this context, heterogeneity has been shown to generate subclones with different genetic and epigenetic profiles within the same primary tumor and its metastases (38, 39).

**FIGURE 4:**
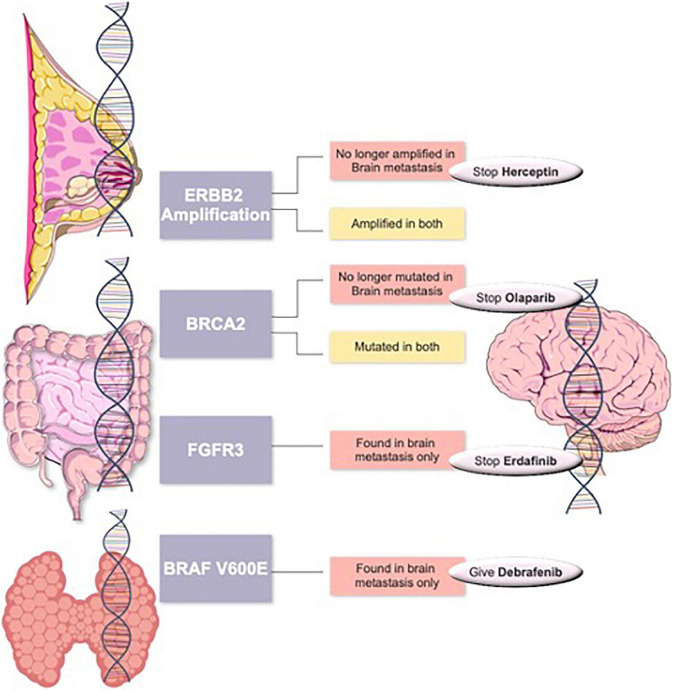
Suggested targeted therapy scheme in primary tumors such as breast cancer, colorectal cancer or thyroid cancers with changed management in case of changes in their brain metastasis tumors.

Routine clinical analysis of brain metastases using Comprehensive Genomic Panels (CGP) can positively impact the survival rate of patients by allowing the administration of pathway-specific therapeutic interventions. Integration and widespread clinical use of NGS or CGP sequencing for such patients, especially in view of positive clinical characteristics of a lower tumor grade and no nodal involvement, could provide a cost-effective alternative for patients who would otherwise suffer the consequences of the debilitating and non-specific classical treatment of chemotherapy. For sample collection, some studies have used the less invasive technique of obtaining tissue samples from CSF, and thus avoiding a resection surgery (35, 40). Such patients will benefit from reassessing their tumors for a better targeted therapy (see examples in Figure 4). We propose the routine use of CGP for brain metastasis patients similar to that described in our previous studies (18, 19, 41).

In summary, we showed that the genetic signature of CNS and systemic metastases can differ from their primary tumor. We also report that clinically actionable genetic alterations present in brain metastases are frequently not detected in primary tumors. Thus, patients only screened for primary tumor mutations could miss the opportunity for a targeted therapy to combat their brain metastases.

## Data availability statement

The datasets presented in this study can be found in online repositories. The names of the repository/repositories and accession number(s) can be found below: https://www.ncbi.nlm.nih.gov/genbank/, TBD.

## Ethics statement

The studies involving human participants were reviewed and approved by King Fahad Medical City IRB 19-501. Written informed consent for participation was not required for this study in accordance with the national legislation and the institutional requirements.

## Author contributions

DB and MAb analysed the data and wrote the manuscript. EA, FA, AAlm, SAlO, and AB performed the NGS experiments and analysed the genetic data. SAA, LA, SAlm, DA, SRA, and MAl collected the clinical data. FAA, WA, and AAla contributed to clinical data collection. HA-H critically reviewed the manuscript. MAb designed and oversaw the study and edited the manuscript. HR performed all statistical analysis and edited the manuscript. All authors approved the final manuscript.
